# Combination of Near Infrared Light-Activated Photodynamic Therapy Mediated by Indocyanine Green with Etoposide to Treat Non-Small-Cell Lung Cancer

**DOI:** 10.3390/cancers9060063

**Published:** 2017-06-05

**Authors:** Ting Luo, Qinrong Zhang, Qing-Bin Lu

**Affiliations:** 1 Department of Physics and Astronomy, University of Waterloo, 200 University Avenue West, Waterloo, ON N2L 3G1, Canada; t3luo@uwaterloo.ca; 2 Departments of Biology and Chemistry, University of Waterloo, 200 University Avenue West, Waterloo, ON N2L 3G1, Canada

**Keywords:** photodynamic therapy (PDT), chemotherapy, lung cancer, combination therapy, indocyanine green (ICG), etoposide (VP-16)

## Abstract

Indocyanine green (ICG) has been reported as a potential near-infrared (NIR) photosensitizer for photodynamic therapy (PDT) of cancer. However the application of ICG-mediated PDT is both intrinsically and physiologically limited. Here we report a combination of ICG-PDT with a chemotherapy drug etoposide (VP-16), aiming to enhance the anticancer efficacy, to circumvent limitations of PDT using ICG, and to reduce side effects of VP-16. We found in controlled in vitro cell-based assays that this combination is effective in killing non-small-cell lung cancer cells (NSCLC, A549 cell line). We also found that the combination of ICG-PDT and VP-16 exhibits strong synergy in killing non-small-cell lung cancer cells partially through inducing more DNA double-strand breaks (DSBs), while it has a much weaker synergy in killing human normal cells (GM05757). Furthermore, by studying the treatment sequence dependence and the cytotoxicity of laser-irradiated mixtures of ICG and VP-16, we found that the observed synergy involves direct/indirect reactions between ICG and VP-16. We further propose that there exists an electron transfer reaction between ICG and VP-16 under irradiation. This study therefore shows the anticancer efficacy of ICG-PDT combined with VP-16. These findings suggest that ICG-mediated PDT may be applied in combination with the chemotherapy drug VP-16 to treat some cancers, especially the non-small-cell lung cancer.

## 1. Introduction

Photodynamic therapy (PDT) is a cancer therapy in which tumor destruction is achieved through the generation of cytotoxic reactive oxygen species (ROS) by exposing the diseased tissue to a photosensitizer and light of an appropriate wavelength. PDT has several advantages over conventional radiotherapy and chemotherapy, including fewer side effects, local targeting, and less acquired resistance to the treatment. Combination of PDT with conventional chemotherapy has been explored as a promising approach for enhancing the antitumor activity of single therapies and reducing side effects associated with the administration of chemotherapeutic drugs. Both additive and synergistic enhancements of cytotoxicity by combination treatments have been reported in some studies [1,2,3,4,5,6,7,8], but antagonistic effects have also been reported [6,8,9]. These results suggest that the combination effects have strong dependence upon a variety of factors including cell type, the photosensitizer, the chemotherapeutic drug under investigation, and the treatment schedule. Moreover, a molecular mechanism for synergistic enhancement of cytotoxicity is often not available.

Indocyanine green (ICG) is a near-infrared fluorescent dye that is approved by the FDA for various clinical applications [10,11,12]. ICG has an absorption maximum near 800 nm; therefore, it can be activated by near-infrared (NIR) light that penetrates relatively deep into tissues. ICG has been considered to be a potential NIR photosensitizer for PDT. The antitumor activity of ICG-PDT has been reported [13,14,15,16,17,18,19,20,21,22]. It has been suggested that the photocytotoxicity of ICG involves the generation of singlet oxygen species (^1^O_2_) [13,15]. However, the yield of ^1^O_2_ has been found to be extremely low due to a low yield of excited triplet states (~10^−5^) [23], which is believed to be responsible for the generation of ^1^O_2_. Moreover, this dye has a short blood half-life of 2–4 min [24], poor photo- and thermal-stability, non-specific binding with proteins, and is vulnerable to aggregation [25]. These properties of ICG have greatly limited its application as a PDT agent.

Etoposide (VP-16) is a semisynthetic podophyllotoxin derivative that is currently in clinical use for the treatment of various cancers [26,27]. It is believed to act on type II topoisomerases (TOPII), which can bind covalently with the 5′ ends of DNA, resulting in the formation of transient double-strand breaks (DSBs). Binding of VP-16 with TOPII can create permanent DNA DSBs that may activate cellular pathways leading to cancer cell death. Although etoposide is efficient, it has severe side effects such as alopecia, gastrointestinal toxicities, leucopoenia, myelosuppression, and the development of secondary leukemia [27,28,29,30,31].

One of the approaches to overcome limitations of chemotherapy drugs is to combine conventional chemotherapy with photodynamic therapy. In PDT, both the photosensitizing agents and light are not toxic by themselves. It is the combination of these two components that produces antitumor effects. PDT can generally reduce the systemic toxic side effects of the chemotherapeutic drug, due to its local targeting and potential synergistic effects that can reduce the required dose of the chemotherapeutic drug, and has the potential to overcome drug resistance via generating new pathways to kill tumor cells. Therefore, researchers have investigated the combination effects of PDT with chemotherapy that involves cisplatin (CDDP), 5-fluorouracil (5-FU), etoposide (VP-16), and a number of other chemotherapeutic drugs [1,2,3,4,5,6,7,8,9,32,33,34,35]. Some of these combinations have been demonstrated to produce synergistic enhancement of cytotoxicity. However, a mechanistic understanding of synergy induced by the combination treatment is still lacking.

In this study, we show in vitro combination anticancer effects of ICG-mediated PDT and chemotherapy mediated by etoposide (VP-16) to treat human non-small-cell lung cancer. We also studied the treatment sequence dependence of the combination effects. Moreover, the generation of DNA DSBs were measured and quantified. Furthermore, a molecular mechanism of action for this combination therapy is proposed.

## 2. Results

### 2.1. Photocytotoxicity of ICG in Treating A549 Cells

**Figure 1:**
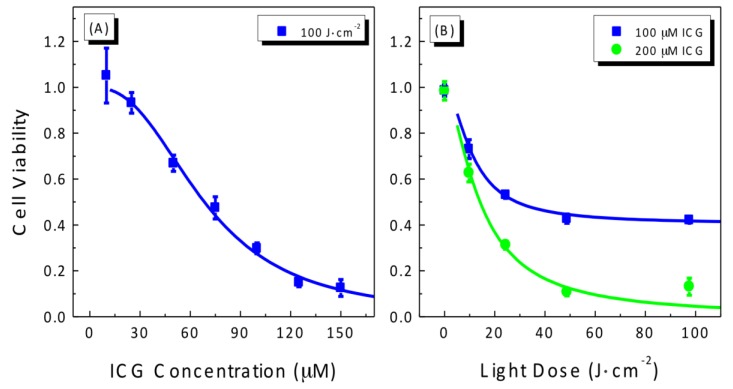
(**A**) Drug-dose and (**B**) light-dose response curves for cell viability of A549 cells treated with Indocyanine green (ICG)-mediated photodynamic therapy (PDT). Cells were incubated with ICG for 5 h and then irradiated at 800 nm. Cell viability was measured by the standard MTT (3-(4,5-dimethylthiazol-2-yl)-2,5-diphenyltetrazolium) survival assay. The solid lines are best fits to the experimental data using a logistic function in Origin software.

We firstly evaluated the photodynamic efficacy of ICG activated by an 800 nm laser. In this experiment, A549 cells were incubated with various concentrations of ICG for 5 h and then exposed to laser light irradiation. Cell viability was determined by using the standard MTT (3-(4,5-dimethylthiazol-2yl)-2,5-diphenyltetrazolium) survival assay described in Section 4.3. The results are shown in Figure 1. Photocytotoxicity of ICG increased in a drug and light-dose dependent manner. At a light dose of 100 J/cm^2^, the IC_50_ of ICG was calculated to be 68.5 ± 1.7 μM. The LD_50_ values were found to be 28.8 ± 1.9 and 14.1 ± 1.2 J/cm^2^ for 100 and 200 μM ICG, respectively.

Anticancer activity of ICG via the photodynamic effect has been studied in a number of cell lines, including HeCaT keratinocytes, HT-29 human colon cancer, SCL1 and SCL2 squamous cell carcinoma, N1 fibroblasts, human SKMEL 188 melanoma, mouse S91 melanoma, human skin Sk-Mel-28 melanoma, and MDA-MB231 breast cancer cells [13,14,15,18,20,21,36]. For example, with 24 h drug-incubation and a light dose of 24 J/cm^2^, the IC_50_ values of ICG were reported to be 20.1, 43.9, 61.8, and 62.3 μM in HaCaT, SCL1, SCL2, and N1 cells, respectively [36]. The PDT efficacy of ICG in A549 cells observed in the present study is consistent with those reported for these cell lines. As stated in Section 4.2, our irradiation experiments of the cells were conducted with the average laser power density of 81 mW/cm^2^ and the durations of exposure of 0–100 J/cm^2^, which were much lower than those used in photothermal experiments. Under these conditions, there was hardly any detectable temperature increase in cells or tissues [37]. Indeed, the photo-cytotoxicity purely via the PDT effect has been proven for low power density of 5–10 W/cm^2^ and exposure times of seconds or minutes [14,36,37]. Moreover, our results in Section 2.2 and Section 2.4 (below) also provide evidence for no or negligible thermal effects involving our current experiments.

However, ICG is much less efficient in killing cancer cells than Photofrin^®^, the first clinically approved and currently the most widely used photosensitizer, which has an IC_50_ value as low as 0.5 μM in A549 cells at a lower light dose [38]. Moreover, the blood half-life of ICG was reported to be only 2–4 min [24]. It will be very difficult to achieve an ICG concentration that is high enough to exhibit significant antitumor effects under in vivo conditions. Therefore, we explored the combination of ICG-PDT with conventional chemotherapy as a potential way to enhance the effectiveness of ICG as a promising NIR agent for PDT.

### 2.2. Photocytotoxicity of ICG-PDT Combined with VP-16 in Treating A549 Cells

**Figure 2:**
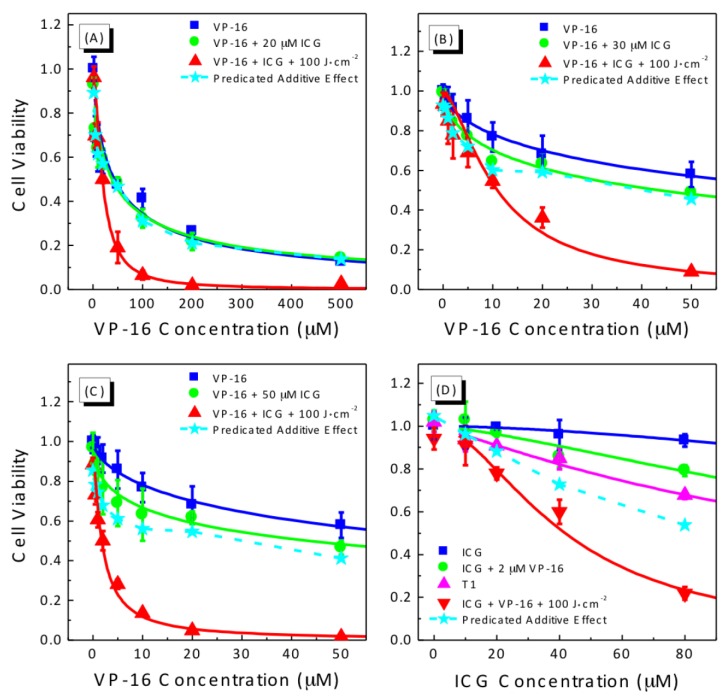
Drug-dose response curves (**A**–**D**) for cell viability of A549 cells treated with the combination of ICG-PDT and VP-16. Cells were treated with ICG and VP-16 for 4 h after overnight incubation and then irradiated with 800 nm laser at a light dose of 100 J/cm^2^. Cell viability was measured by the standard MTT assay. The solid lines are best fits to the experimental data using a logistic function in Origin software. The dashed line and solid stars represent the calculated additive effect.

**Figure 3:**
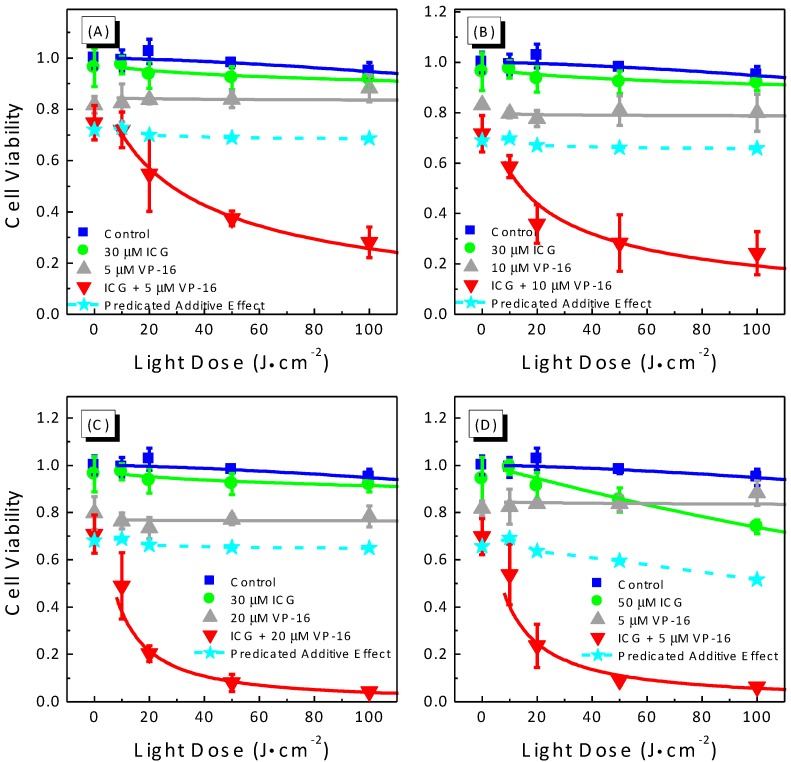
Light-dose response curves (**A**–**D**) for cell viability of A549 cells treated with the combination of ICG-PDT and VP-16. Cells were treated with ICG and VP-16 for 4 h after overnight incubation and then irradiated with an 800 nm laser at a light dose of 100 J/cm^2^. Cell viability was measured by the standard MTT assay. The solid lines are best fits to the experimental data using a logistic function in Origin software. The dashed lines and filled stars represent the calculated additive effect.

We measured drug- and light-dose responses of A549 cells to the combination treatment of ICG-PDT and VP-16 (Figure 2 and Figure 3).

As shown in Figure 2A, with a drug incubation time of 4 h, IC_50_ of VP-16 was determined to be 41.7 ± 7.7 μM in A549 cells. When combining VP-16 with 20 μM ICG, cytotoxicity was not affected significantly (IC_50_ = 37.4 ± 8.7 μM). The addition of laser irradiation, at a light dose of 100 J/cm^2^, reduced the IC_50_ dramatically to 18.8 ± 1.6 μM, which was ~45% of that of VP-16. With higher ICG concentrations of 30 (Figure 2B) and 50 μM (Figure 2C), a slight increase in cytotoxicity was observed even without laser irradiation. Within the range of drug concentrations in Figure 2B,C, the IC_50_ values for VP-16/VP-16 + ICG without irradiation could not be determined accurately. At a light dose of 100 J/cm^2^, the values of IC_50_ were calculated to be 10.9 ± 0.7 and 1.77 ± 0.17 μM, which were 26% and 4% of the IC_50_ of VP-16, for the addition of 30 and 50 μM ICG, respectively.

We also investigated the dependence of cytotoxicity of the combination treatment on ICG concentration (Figure 2D). ICG was found to be only slightly cytotoxic in A549 cells with less than 7% of cells killed up to a concentration of 80 μM. VP-16 alone at a concentration of 2 μM was found to be not cytotoxic. Without irradiation, the combination of 2 μM VP-16 with ICG at low concentrations (0–20 μM) did not show enhanced cytotoxicity, while at higher ICG concentrations of 60 and 80 μM, cytotoxicity was increased from 4.1% ± 7.0% to 14.3% ± 4.7% and from 6.7% ± 2.9% to 20.7% ± 2.6%, respectively. With laser irradiation, the IC_50_ of ICG was found to be 155.7 ± 15.3 μM. In the presence of 2 μM VP-16, the IC_50_ was reduced by more than 73% (41.8 ± 1.8 μM). Moreover, we performed fractional effect analysis to evaluate the combination effects as described in Section 4.8. Considering the dark cytotoxicity of ICG + VP-16, we compared the combination effects with the cytotoxicity of ICG + VP-16 instead of that of VP-16 alone. As shown in Figure 2, the dashed line and solid stars represent the calculated predicated additive effects. The observed effects were found to be the equal to or lower than the predicated additive effects, indicating additive to synergistic enhancement of cytotoxicity induced by the combination treatment.

We observed a wide range of synergistic enhancements of cytotoxicity by the combination treatment of ICG-PDT and VP-16 and the degree of synergy had a strong dependence upon the concentration of both ICG and VP-16. Although we also observed slight increases in cytotoxicity in cells treated with ICG and VP-16 without laser irradiation, this effect requires relative high concentrations of ICG and VP-16. It is well known that the blood half-life of ICG is about 2–4 min [24], resulting in limited accumulation of ICG in tissues and organs. Therefore, the dark effect is not likely to be significant under in vivo conditions. Moreover, one objective of this study was to overcome the severe side effects of VP-16. Thus, it was considered to be desirable to lower the concentration of VP-16, which is also expected to help reduce dark cytotoxicity of the combination treatment.

We also performed experiments to study the light-dose dependence of cytotoxicity produced by the combination treatment of ICG-PDT and VP-16. Results are shown in Figure 3. As discussed above, low concentrations of both ICG and VP-16 should be used to minimize the dark cytotoxicity induced by the combination of ICG and VP-16. In this experiment, we varied the irradiation time to achieve various light doses between 0 and 100 J/cm^2^ and evaluated the cytotoxicity of several combinations of ICG and VP-16. As shown in Figure 3, laser irradiation in the absence of both drugs was not cytotoxic up to a light dose of 100 J/cm^2^. Cytotoxicity of VP-16 alone did not change significantly with increasing light dose (Figure 3A–C). At a concentration of 30 μM, ICG did not show significant photocytotoxicity (Figure 3A). When cells were incubated with 50 μM ICG (Figure 3D), percentages of cells killed were increased from 2.1% ± 1.6% to 14.7% ± 5.1% and from 5.1% ± 3.4% to 26.1% ± 2.9%, at light doses of 50 and 100 J/cm^2^, respectively. The LD_50_ of ICG was much higher than 100 J/cm^2^ and could not be determined accurately from this experiment. These results indicate no or little photothermal effect of ICG under the current irradiation conditions. With laser irradiation, cell viability of the groups incubated with both ICG and VP-16 decreased dramatically with increasing light dose; and the LD_50_ values were determined for different combinations: 28.0 ± 9.3, 14.4 ± 10.4, 6.55 ± 2.79 J/cm^2^ for 30 μM ICG with 5, 10, and 20 μM VP-16, and 6.84 ± 6.06 J/cm^2^ for 50 μM ICG with 5 μM VP-16.

In addition, we performed fractional effect analysis and the expected additive effects were plotted as dashed lines and filled stars in Figure 3. With laser irradiation, the observed combination effects were found to lie below the additive effect curves for all the concentrations of ICG and VP-16 evaluated in this experiment, confirming a synergistic enhancement of cytotoxicity by the combination treatment of ICG-PDT and VP-16 in a wide range of drug concentrations and light doses.

The application of ICG as a PDT agent has been limited by the short blood half-life of 2–4 min [24], which lowers the accumulation of this drug in tissues and organs. In this study, we found that at a light dose of 100 J/cm^2^ (typical light dose range for clinic applications of conventional PDT: 25–500 J/cm^2^ [39]), IC_50_ of ICG is much higher than 80 μM in A549 cells. In cells treated with 50 μM ICG and laser irradiation, LD_50_ is much higher than 100 J/cm^2^. These results indicate that relative high drug- and light-doses are required for effective killing of cancer cells by ICG alone. Here, we demonstrated that the co-incubation of A549 cells with ICG and low dose VP-16 can increase the cytotoxicity in a synergistic manner leading to reduced effective doses of both drugs as well as light doses. A more than 20 times reduction in IC_50_ of VP-16, from >40 μM to 1.77 μM, was observed in cells treated with VP-16 and 50 μM ICG. Moreover, we found that the degree of enhancement is light-dose dependent. These observations indicate that combining PDT mediated by ICG and VP-16 chemotherapy is a promising approach for improving the therapeutic effectiveness of ICG-PDT and reducing the side effects of VP-16 chemotherapy at the same time.

### 2.3. Photocytotoxicity of ICG-PDT Combined with VP-16 in Treating GM05757 Cells

**Figure 4:**
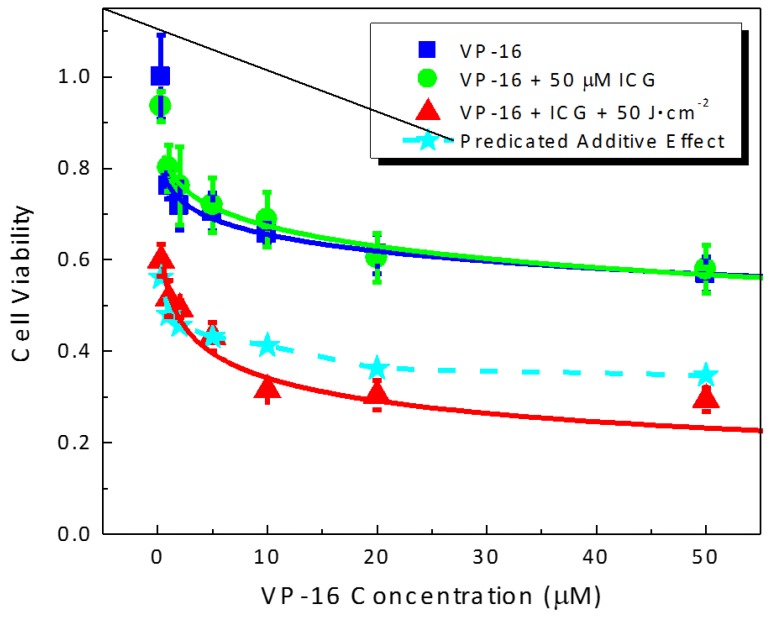
Drug-dose response curves for cell viability of human normal cells (GM05757) treated with the combination of ICG-PDT and VP-16. Cells were treated with ICG and VP-16 for 4 h after overnight incubation and then irradiated with an 800 nm laser at a light dose of 100 J/cm^2^. Cell viability was measured by the standard MTT assay. The solid lines are best fits to the experimental data using a logistic function in Origin software. The dashed line and filled stars represent the calculated additive effect.

We tested the toxicity of the combination treatment of ICG-PDT and VP-16 chemotherapy in a human normal cell line GM05757. The results are shown in Figure 4. Sensitivity to VP-16 chemotherapy treatment was found to be similar in GM05757 normal fibroblast and A549 lung cancer cells with a ~40% cell killing effect observed at a VP-16 concentration of 50 μM in both cell lines. No significant difference was observed between the groups treated with VP-16 alone or VP-16 + 50 μM ICG. At an ICG concentration of 50 μM and a light dose of 50 J/cm^2^, cell viabilities were 60.0% ± 3.5% and 85.3% ± 5.1% in GM05757 and A549 cells, respectively. This result suggested that GM05757 cells were more sensitive than A549 cells to ICG-PDT treatment. When treated with 50 μM ICG and irradiated at 50 J/cm^2^, there were still 29.5% ± 2.6% of the cells survived at a VP-16 concentration of 50 μM in GM05757 cells (Figure 4). In contrast, cell viability was found to be 9.2 ± 1.8% at a much lower VP-16 concentration of 5 μM in A549 cells (Figure 3D), indicating a much stronger synergy. Although the combination effects were found to be lower than the expected additive effects, the degree of synergy was much lower in GM05757 cells than that observed in A549 cells.

Results in this experiment showed that the normal cells may have similar levels of sensitivity to PDT treatment as those of cancer cells. Fortunately, in PDT, local targeting of the diseased tissue can be achieved by selective delivery of laser beams. Sensitivity to VP-16 treatment has been found to be similar in GM05757 and A549 cells. However, the combination treatment can induce a much stronger synergistic effect in A549 cells than in GM05757 cells and is more effective in killing A549 cells than GM05757 cells.

### 2.4. Sequence Dependence of the Cytotoxicity Induced by ICG-PDT Combined with VP-16

**Figure 5:**
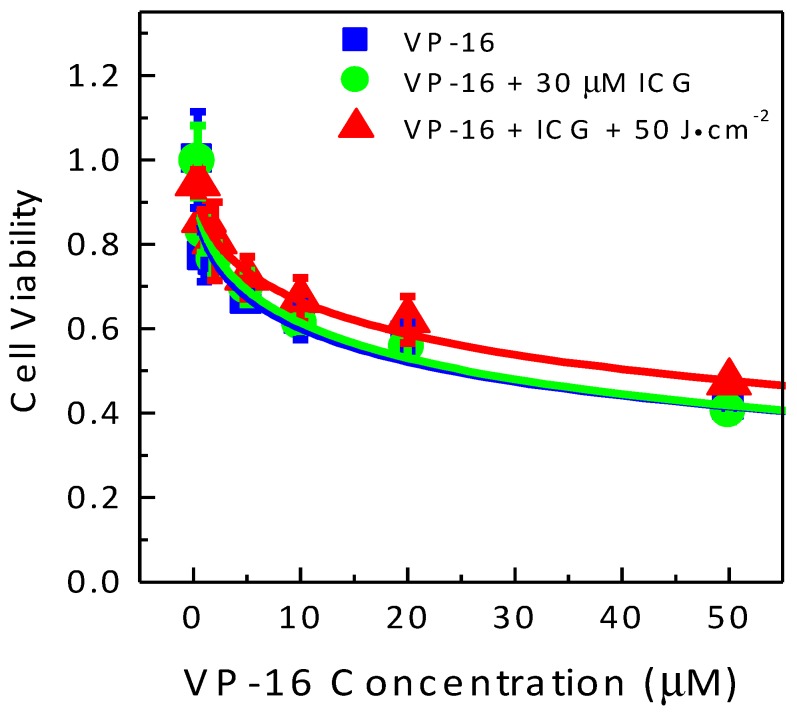
Sequence-dependent cytotoxicity of the combination treatment of ICG-PDT and VP-16 in A549 cells. Cells were incubated with ICG for 4 h and then irradiated at 800 nm. VP-16 was added after laser irradiation. Cell viability was measured by the standard MTT assay. The solid lines are best fits to the experimental data using a logistic function in Origin software.

Whether or not the presence of VP-16 during laser irradiation was required for synergistic enhancement of cytotoxicity was investigated. In this experiment, A549 cells were incubated with ICG for 4 h and then irradiated at 800 nm. After laser irradiation, cells were incubated with VP-16 for 4 h. MTT assay was performed 45 h after the removal of VP-16. The results are shown in Figure 5.

At the highest tested VP-16 concentration 50 μM, we observed a cell viability of ~50% in A549 cells; it was similar to the level of cytotoxicity observed in Section 2.2, where cells were treated with VP-16 before laser irradiation. No significant difference in cell viability was observed among the groups treated with VP-16, VP-16 + ICG, and VP-16 + ICG + 50 J/cm^2^ in A549 cells. This is in contrast to the strong synergistic effects observed in cells co-incubated with ICG and VP-16 before laser irradiation. This observation suggested that the presence of VP-16 at the time of laser irradiation was essential for synergistic enhancement of cytotoxicity induced by the combination treatment of ICG-PDT and VP-16.

### 2.5. Cytotoxicity of Laser-Irradiated Mixtures of ICG and VP-16

**Figure 6:**
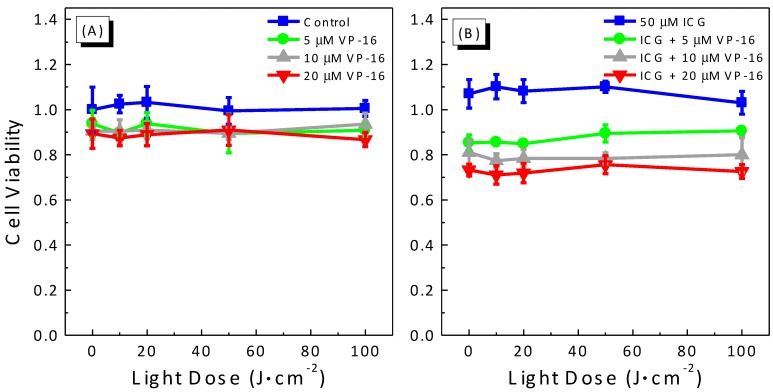
Cytotoxicity of laser-irradiated mixtures of ICG and VP-16 in A549 cells. Cells were treated with (**A**) photo-irradiated VP-16 in cell culture medium and (**B**) photo-irradiated mixtures of ICG and VP-16 in cell culture medium for 4 h. Cell viability was evaluated by using the standard MTT assay.

This experiment was performed to test the cytotoxicity of laser-irradiated mixture of ICG and VP-16. Several mixtures of 50 μM ICG with 5, 10 and 20 μM VP-16 were prepared in the complete cell culture medium and they were irradiated with various light doses. Cells were incubated with the laser-irradiated mixtures of drugs for 4 h and then incubated with drug-free medium for 45 h before adding MTT. The results are shown in Figure 6.

Cytotoxicity of the cell culture medium and VP-16 at all three concentrations evaluated in this experiment did not show significant dependence on light dose (Figure 6A). Similarly, in cells treated with the mixtures of 50 μM ICG and VP-16, cytotoxicity did not change significantly with increasing light dose (Figure 6B). When ICG was combined with VP-16, we noted that the cell viability decreased slightly compared with the groups treated with VP-16 alone. This was attributed to the dark effect discussed in Section 2.1. These results showed that the cytotoxicity of the combination treatment of ICG-PDT and VP-16 differed dramatically from that of the laser-irradiated mixtures of the two drugs. Therefore, the observed synergistic effects induced by the combination treatment should involve certain intracellular components such as enzymes.

### 2.6. In Vitro DNA Double-Strand Breaks Meausrement

**Figure 7:**
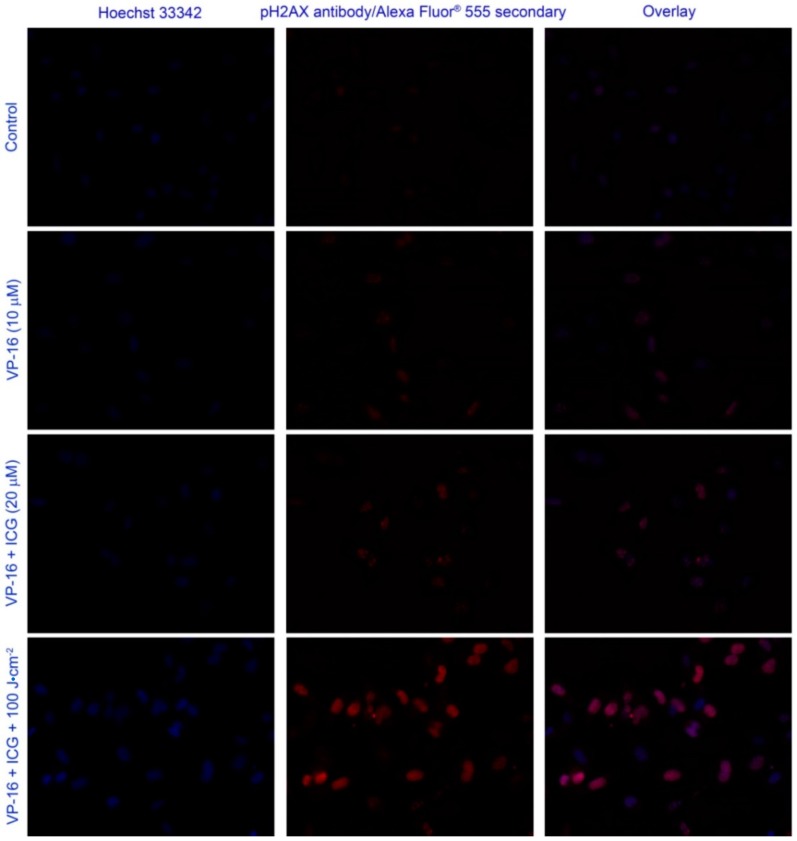
Representative images of the combination treatment of ICG-PDT and VP-16 in A549 cells using the HCS DNA damage kit. The cells were treated with VP-16 and ICG after overnight incubation. After 4 h incubation, the cells were washed twice with PBS and irradiated at 800 nm. DNA damages were measured using the HCS DNA damage kit. The images were taken on a Nikon Eclipse TS100/TS100-F microscope Alexa Fluor^®^ 555 (Invitrogen, Life Technologies Inc., Burlington, Canada) is shown in red ($\lambda_{ex}$ = 510–560 nm; $\lambda_{em}$ > 590 nm). Hoechst 33342 is shown in blue ($\lambda_{ex}$ = 330–380 nm; $\lambda_{em}$ > 420 nm). Background images were taken under the same experimental conditions and subtracted using Photoshop software.

To further investigate the mechanism underlying the synergistic effects observed in MTT assay results, we measured the yield of in vitro DNA DSBs. It is well known that DNA DSBs are potent inducers of mutations and of apoptosis [40,41]. The commercial HCS DNA damage kit was used. Representative images of treated A549 cells are shown in Figure 7. VP-16 is known to cause DNA DSBs and γH2AX has been shown to be a sensitive marker of DNA DSBs induced by a variety of DNA damaging agents including VP-16 [42,43,44,45]. In the present study, γH2AX foci (red channel) were indeed observed when cells were treated with VP-16 (row #2). While the brightness of red fluorescence did not change significantly when cells were treated with VP-16 and ICG (row #3), laser irradiation produced significantly more γH2AX foci as shown in the last row.

**Figure 8:**
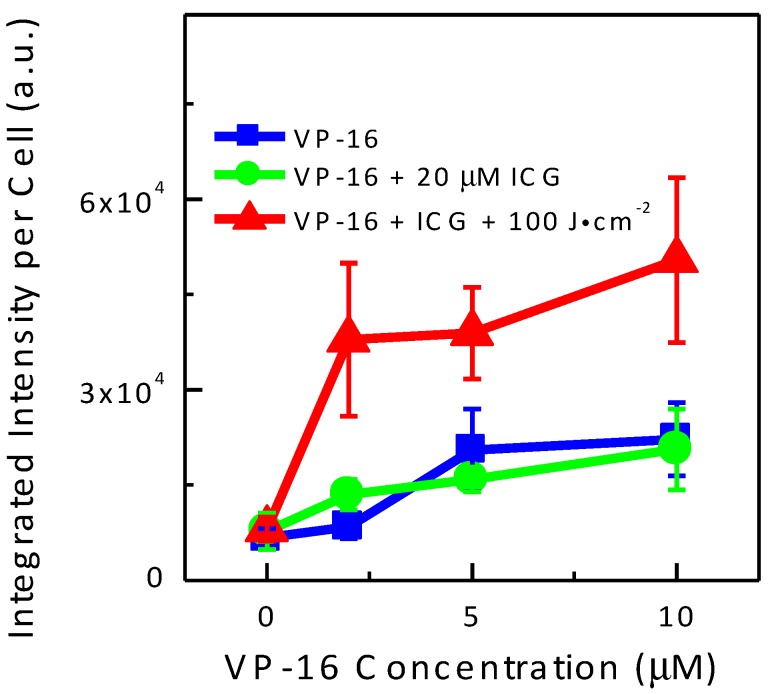
Integrated Alexa Fluor^®^ 555 fluorescence intensity per cell as a function of VP-16 concentration in A549 cells using the HCS DNA damage kit. The integrated fluorescence intensity was calculated and the number of cells was counted using the Photoshop software. Results represent mean ± S.D. of at least three images.

It has been suggested that the relative yield of DNA DSBs could be measured more accurately by using the total area of γH2AX foci than the total number of the foci [42]. Therefore, we plotted the average of integrated fluorescence intensity of the red channel as a function of VP-16 concentration in Figure 8. Intensity of γH2AX foci increased with increasing VP-16 concentration. When cells were treated with ICG and VP-16, the intensity of γH2AX foci was not changed significantly in A549 cells. These results indicated that increased generation of DNA DSBs by the combination treatment of ICG-PDT and VP-16 was at least partially responsible for the synergistic enhancement of cytotoxicity observed in MTT experiments.

## 3. Discussion

ICG was chosen in this study for several reasons. First, The FDA has approved the clinical applications of ICG other than PDT. Second, ICG-PDT is activated by NIR light that can penetrate deeper into tissues. Therefore, the combination of ICG-PDT with VP-16 has the potential of treating larger tumor volumes. Combination of PDT with VP-16 was reported by Gantchev et al. [5,46]. However, the photosensitizers used in those studies are metallic phthalocyanine (AlPcS4/ZnPcS4), which requires red light for activation (λ_max_ ~670 nm [47]). Stolik et al. [48] reported that the penetration depths of 780 and 835 nm light are ~10–130% larger than that of 674 nm light depending on tissue type. In lung carcinoma, for example, the values were determined to be 2.01, 2.82, and 3.89 mm for 674, 780, and 835 nm light, respectively. Therefore, ICG-mediated PDT has the potential of treating deeper tumors than those that can be treated by AlPcS4/ZnPcS4-mediated PDT. Third, there have been tremendous efforts on improving the tumor specificity of ICG as well as increasing accumulation of the drug into tumors and some of these systems have been shown to be superior to free ICG as imaging and photothermal therapy agents [49,50,51,52,53,54,55]. It would be of great interest to explore the PDT effectiveness of those systems either alone or in combination with other treatment modalities such as VP-16-mediated chemotherapy.

In this study, we performed in vitro cytotoxicity studies of the combination effects of ICG-PDT with VP-16 on the human non-small-cell lung cancer (A549) cell line and the human normal fibroblast (GM05757) cell line. Sensitivity to VP-16 treatment was found to be similar between A549 and GM05757 cells. GM05757 cells were shown to be more sensitive to ICG-PDT treatment than A549 cells. With combination treatment of ICG-PDT and VP-16, strong synergistic enhancement of cytotoxicity was observed in A549 cells in wide ranges of drug concentrations and light doses. A more than 95% reduction in IC_50_ was obtained in cells co-treated with VP-16 and 50 μM ICG + 100 J/cm^2^. The degree of enhancement was found low in GM05757 cells. We measured the relative yields of DNA DSBs by using the commercial HCS DNA damage kit and found that the combination treatment can increase the yields of DSBs by ~2-fold in A549 cells. These findings suggest that ICG-mediated PDT may be combined with VP-16 in treating lung cancers. Synergistic enhancement of cytotoxicity has the potential to enhance the effectiveness of treatment and more importantly, to reduce side effects of the chemotherapeutic drugs. Furthermore, the observation of a strong dependence of the degree of synergy on light dose suggests that the reaction can be controlled by light. Targeted delivery of laser beams may further reduce the overall toxicity of the treatment. In addition, the synergism was demonstrated to depend on the sequence of treatment. Synergistic effects were not observed when VP-16 was added after laser irradiation. This finding indicates the involvement of direct or indirect interaction between ICG and VP-16 during the course of laser irradiation. We also evaluated the cytotoxicity of the laser-irradiated mixtures of the drugs and found that intracellular components, probably enzymes, might be required for the activation of the drugs.

Previously, Gantchev et al. suggested that the mechanism for synergistic enhancement of cytotoxicity produced by the combination of metallic phthalocyanine-mediated PDT and VP-16 is PDT-induced depletion of intracellular glutathione (GSH), an antioxidant that is believed to be able to reduce the highly cytotoxic radicals of VP-16 [46]. In order to see whether this mechanism is responsible for the synergism observed in the present study, we performed an experiment in which the cells were treated with VP-16 immediately after laser irradiation (see Section 2.4). We would have observed synergistic enhancement of cytotoxicity, if the ICG-PDT treatment induced cellular damages, which were expected to last for hours following laser irradiation [21], had potentiated the cytotoxicity of VP-16. In contrast, the results in Figure 5 indicate that the strong synergy induced by the combination of ICG-PDT and VP-16 is not likely due to ICG-PDT-produced cellular damages but should involve direct or indirect interaction between ICG and VP-16 during the course of laser irradiation.

Based on the following considerations, we here propose a mechanism for obtained results in the present study: the enzyme-mediated electron-transfer reaction from VP-16 to ^1^ICG* could induce synergistic enhancement of cytotoxicity in vitro. First, we calculated the free energy change of the electron-transfer reaction between ICG/^1^ICG* and VP-16 by using the following Rehm-Weller Equation (1) [56]:

(1) $$∆G\left(eV\right)=nF\left(E_{ox}-E_{red}\right)-w_{p}-E_{00}$$

In this equation, *n* is the number of electrons transferred, *F* is the Faraday’s constant, *E_ox_* and *E_red_* are the oxidation potential of the donor and reduction potential of the acceptor, respectively, $w_{p}\;$describes the Coulombic attraction between ions generated by electron transfer reactions, and *E*_00_ is the energy of excitation. For most electron transfer reactions, *nF* is approximately equal to one and can be disregarded in the calculations. The work term $w_{p}$, can also be disregarded due to the lack of electrostatic attraction between a charged species (ICG) and a neutral species (VP-16). The oxidation potential of VP-16 is 0.51 eV vs. SHE (standard hydrogen electrode) at pH 7 [57]. The reduction potentials of ICG in acetonitrile have been reported to be −0.59 eV vs. SHE [58]. Therefore, the free energy change of one-electron transfer from ICG to VP-16 can be estimated to be 1.1 eV suggesting that this reaction is thermodynamically unfavorable. However, when ICG is excited to ^1^ICG*, the free energy change can be estimated to be −0.45 eV. A negative free energy change indicates that one-electron transfer from VP-16 to ^1^ICG* is thermodynamically favorable. Second, we demonstrated that incubating the cells with VP-16 immediately after ICG-PDT treatment did not show any synergistic effect. This observation cannot be explained by independent actions of ICG-PDT and VP-16 to activate different cellular pathways. Third, the binding of VP-16 with intracellular enzymes such as oxidases may lower the activation energy and increase the reaction rate. In the human body, VP-16 can be oxidized by oxidases to phenoxy radicals [47,59], which may be further converted to metabolites that have been shown to be more reactive than the parent compound VP-16 in inducing DNA DSBs [60,61,62,63]. Therefore, ^1^ICG* may act as an electron acceptor in those systems and facilitate the generation of VP-16 radicals.

Based on these results, we propose an electron-transfer-based mechanism for synergistic effects observed in cytotoxicity studies.

This electron-transfer-based combination of ICG-PDT and chemotherapy should not be limited to that with VP-16. Recently, Lu et al. [64] obtained the precise molecular mechanism of action for the chemotherapeutic drug cisplatin (CDDP) and proposed that the dissociative electron-transfer (DET) reaction between CDDP and the guanine base in DNA, which is most likely to donate an electron among the four DNA bases, is responsible for the activation of CDDP. In another study, Lu [65] demonstrated that one-electron transfer from the excited singlet state of indocyanine green (^1^ICG*) to CDDP can increase the yield of DNA DSBs in plasmid DNA and suggested that the combination of these two drugs may produce enhanced cytotoxicity against cancer cells. Based on this electron-transfer mechanism, we developed a combination therapy of CDDP with a biological electron donor (TMPD; N,N,N′,N′-tetramethyl-p-phenylenediamine), which can enhance the cytotoxicity of CDDP in a synergistic manner and lead to a complete removal of cisplatin-resistance in the human lung A549 and ovarian NIH:OVCAR-3 cancer cells [66]. These findings suggest that the electron-transfer reaction between ^1^ICG* and CDDP may have application in cancer treatment as well. More importantly, since ICG is activated by light, selective initiation of the electron-transfer reaction may be achieved by controlled delivery of light and thus, lead to further reduction in toxic side effects of CDDP.

In summary, these studies may help improve our understanding of the molecular mechanisms underlying the synergistic enhancement of ICG-PDT with chemotherapeutic drugs and thus, facilitate the development of more effective treatment approaches for cancers.

## 4. Materials and Methods

### 4.1. Chemicals, Cell line, and Cell Culture Conditions

The compounds 4,5-Benzoindotricarbocyanine (indocyanine green; ICG; C_43_H_47_N_2_NaO_6_S_2_; MW = 775 g∙mol^−1^) and 4′-demethylepipodophyllotoxin-9-(4,6-o-ethylidene-b-d-glucopyranoside) (etoposide; VP-16; C_29_H_32_O_13_; MW = 588.6 g∙mol^−1^) were purchased from Sigma-Aldrich (Sigma-Aldrich Canada Ltd., Oakville, ON, Canada) and used without any further purification. Stock solution of 5 mM ICG was prepared in ultrapure water (Barnstead Nanopure, Thermo Scientific, Dubuque, IA, USA) and stored in dark at −20 °C. Stock solution of 50 mM VP-16 was made in dimethyl sulfoxide (DMSO) and stored at −20 °C.

The human non-small-cell lung cancer cell line (A549, ATCC#: CCL-185™) was obtained from American Type Culture Collection (ATCC, Manassas, VA, USA). Nutrient mixture F12 Ham Kaighn’s modification (F12K), trypsin-EDTA (0.5 g/L porcine trypsin and 0.2 g/L EDTA·4Na in Hank’s Balanced Salt Solution with phenol red), and penicillin streptomycin antibiotics (PS) were purchased from Sigma-Aldrich (Sigma-Aldrich Canada Ltd., Oakville, ON, Canada), and fetal bovine serum (FBS) was purchased from Hyclone Laboratories (Logan, UT, USA). A549 cells were cultivated in F12K supplemented with 10% FBS, 100 units/mL penicillin G and 100 μg/mL streptomycin. The cell culture was kept at 37 °C in a humidified atmosphere containing 5% CO_2_.

### 4.2. Laser Treatment Conditions

Irradiation was carried out by using an 800 nm laser (pulse duration 120 fs, and pulse repetition rate 1k Hz). The average power was 360 mW and the corresponding average irradiation was 81 mW/cm^2^. Duration of exposure was varied to get different light doses (J/cm^2^).

### 4.3. MTT Cell Survival Assay

Cell viability was evaluated by the standard MTT assay using a commercial kit (V-13154, Invitrogen, Lift Technologies Inc., Burlington, ON, Canada). The stock solution of 12 mM MTT (3-(4,5-dimethylthiazol-2-yl)-2,5-diphenyltetrazolium bromide; MW = 414 g·mol^−1^) was prepared by adding 1 mL of sterile PBS to one 5 mg vial of MTT. The SDS-HCl solution was prepared by adding 10 mL of 0.01 M HCl to 1 mg of SDS (sodium dodecyl sulfate; MW = 288 g·mol^−1^).

At the end of incubation, the medium was replaced with 100 (96-well plates) or 30 (384-well plates) μL of fresh phenol red-free complete culture medium and 10 (96-well plates) or 3 (384-well plates) μL of the MTT solution (5 mg/mL). After 2–4 h incubation, 100 (96-well plates) or 30 (384-well plates) μL of SDS-HCl was added to each well. After 4–18 h incubation, the absorbance at 570 nm was measured with a Multiskan GO microplate spectrophotometer (Thermo Scientific, Mississauga, ON, Canada). Cell viability was expressed as the percentage of the control cells, and the data were presented as means of at least three wells ± S.D (standard deviation).

### 4.4. Photocytotoxicity of ICG in Treating A549 Cells

Exponential growing A549 cells were seeded into 96-well microplates at a density of 5 × 10^3^ cells/well. After overnight incubation, the cells were incubated with ICG for 5 h in the dark. Cells were then washed twice with phosphate buffered saline (PBS) and fresh medium was added for irradiation. After that cells were placed back into the incubator and cell viability was evaluated 20 h later by the standard MTT assay.

### 4.5. Cytotoxicity of ICG-PDT Combined with VP-16

Exponentially growing A549 and GM05757 cells were seeded into 384-well microplates at the same density of 3 × 10^3^ cells/well. After overnight incubation, the cells were incubated with various concentrations of ICG and VP-16 for 4 h in the dark. Cells were then washed twice with PBS and fresh complete culture medium was added before irradiation. The irradiated cells were then kept in an incubator for 45 h and cell viability was evaluated by the MTT assay.

### 4.6. Sequence Dependence of Cytotoxicity Induced by ICG-PDT Combined with VP-16

Exponentially growing A549 cells were seeded into 384-well microplates at a density of 3 × 10^3^ cells/well. Cells were incubated with 50 μM of ICG for 4 h, washed twice with PBS, and fresh complete culture medium was added for irradiation. After laser irradiation, various concentrations of VP-16 were added. After 4 h incubation, cells were washed twice with PBS, fresh complete medium was added, and cells were then put back into an incubator. Cell viability was evaluated by the MTT assay.

### 4.7. Cytotoxicity of Laser-Irradiated Mixtures of ICG and VP-16

Exponentially growing A549 cells were seeded into 384-well microplates at a density of 3 × 10^3^ cells/well. Mixtures of various concentrations of ICG and VP-16 were made in complete F12K medium and then exposed to laser irradiation. The drug mixtures were then added to corresponding wells in 384-well microplates. After 4 h incubation, cells were washed twice with PBS and fresh complete culture medium was added. The cells were then put back into an incubator and cell viability was evaluated 45 h later by the MTT assay.

### 4.8. In Vitro DNA Double-Strand Breaks Measurement

Phosphorylation of histone 2AX (H2AX), a member of the histone 2A family is a marker of DNA double-strand breaks (DSBs). The HCS DNA damage kit (Invitrogen, Life Technologies Inc., Burlington, ON, Canada) was used to measure the yield of DNA DSBs induced by the combination treatment of ICG-PDT and VP-16. A549 cells were seeded and treated with drugs and laser irradiation as described in Section 4.5. At the end of the incubation period (18 h), cells were fixed, permeabilized, and stained following the manufacturer’s protocol without further modification. The images were taken on a Nikon Eclipse TS100/TS100-F microscope with filter sets of *Ex*/*Em* of BP510–560/LP590 nm and BP330–380/LP420 nm for Alexa Fluor^®^ 555 and Hoechst 33342, respectively. All the images were taken with an exposure time of 6 s. Background images were taken for each channel under the same experimental conditions. The final images presented are corrected images obtained after subtracting the background images using Photoshop software (Adobe Systems Inc., San Jose, CA, USA).

The average of integrated fluorescence intensity from the Alexa Fluor^®^ 555 channel was used to quantify the yield of DNA DSBs and is plotted as a function of VP-16 concentration. Each data point represents the mean ± S.D. of three to five images.

### 4.9. Data Analysis

The drug- and light-dose response curves were fitted with a sigmoidal logistic function by using the Origin software (OriginLab, Northampton, MA, USA). The drug concentration (IC_50_) or light dose (LD_50_) required to produce a 50% cell killing effect was determined on the basis of the fitted data.

A modified fractional effect method was used to perform synergy analysis. In this study, we observed that the addition of ICG at non-cytotoxic concentrations could slightly enhance the cytotoxicity of VP-16 even when cells were not exposed to laser irradiation. Considering this “dark effect” of the combination treatment, we modified the fractional effect method for calculating the expected additive effects by using the following Equation (2), in which the effect induced by the chemotherapeutic drug was replaced by the dark effect of ICG and VP-16:

(2) $$f_{com.}=f_{dark}\times f_{PDT}$$

In the formula above, $f_{com.}$, $f_{dark}$, and $f_{PDT}$ denote the fractions of survived cells with the combination (ICG-PDT + VP-16), dark (ICG + VP-16), and PDT (ICG + laser irradiation) treatments, respectively. The observed effects of the combination treatment were then compared with the expected additive effects: the effect was synergistic if the observed cell viability was lower than the expected additive effect, and the effect was antagonistic if it was higher than the calculated value.

## 5. Conclusions

In this article, we have demonstrated that the combination of ICG-PDT with etoposide (VP-16) can synergistically kill non-small-cell lung cancer cells (A549); partially because this combination induces more DNA double-strand breaks (DSBs). However, this combination exhibits a much weaker synergy in killing normal cells (GM05757). Direct/indirect reactions between ICG and VP-16 are involved in the observed synergy, which has been demonstrated through the study of treatment sequence dependence and the study of laser-irradiated mixtures’ cytotoxicity. Based on the results, an electron-transfer-based mechanism has been proposed for synergistic effects observed in these cytotoxicity studies.
